# Demersal and pelagic species of fish and squid from the Patagonian shelf

**DOI:** 10.3897/zookeys.668.11826

**Published:** 2017-04-13

**Authors:** Elena Beatríz Eder, María Rosa Marin, Mirtha Noemí Lewis

**Affiliations:** 1 Centro para el Estudio de Sistemas Marinos CESIMAR-CENPAT-CONICET, Boulevard Almirante Brown 2915, U9120ACD, Puerto Madryn, Chubut, Argentina

**Keywords:** Occurrence, Patagonia, teleosts, elasmobranches, cephalopods, Southwestern Atlantic Ocean, demersal habitat, pelagic habitat

## Abstract

The dataset contains 2007 records of occurrence of 39 species of fish and 2 species of squid distributed on the Patagonian continental shelf and slope. This dataset describes a new and revised version of the original data published through OBIS with individual morphometrics. Specimens are representative of pelagic, demersal, demersal-pelagic, demersal-benthic and benthic habits and they were collected by commercial fishing vessels in autumn (May–June, 2001, 51 catches), winter (July–August, 2001, 38 catches) and summer (January-February, 2002, 112 catches). The sampling was carried out with bottom trawls at a depth range of 73–370 m. The survey was located between 39°–52°S and 55°–65°W.

## Data published through

GBIF: http://arobis.cenpat-conicet.gob.ar:8081/resource?r=argentina-fishes

## Introduction

The Patagonian continental shelf, in the Atlantic margin of South America, is characterized by a remarkable productivity accounted by its oceanographic and bathymetric features, promoting ‘hotspots’ of biodiversity and attracting marine top predators, such as sea birds and marine mammals. The objective of this dataset is to provide basic information (location and depth capture, and individual morphometrics) of potential prey species of marine top predators from the Patagonian shelf, representative of pelagic, demersal, demersal-pelagic, demersal-benthic and benthic habits as useful information for ecological and biogeographical studies.

## Taxonomic coverage

**Figure 1.**
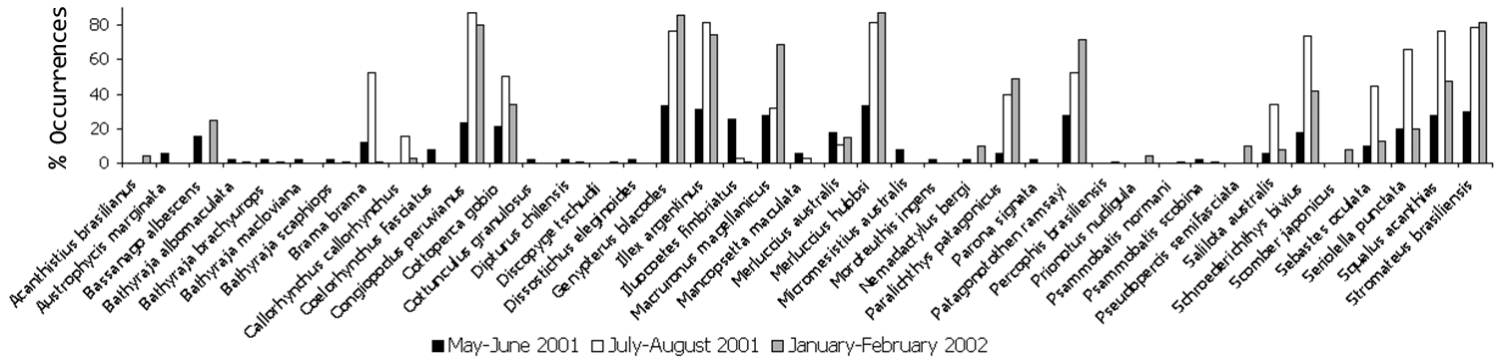
Distribution of occurrences of species during three seasons. Occurrence was calculated in each season as: (number of localizations where the species was present/ total number of localizations)*100.

**General taxonomic coverage description**: All specimens were identified to species level. The dataset included 39 marine species of fish, representative of half the best-known fish species from the Argentine continental shelf (55.7 %, Cosseau and Perrota 2013), and 2 species of squid, that were captured during three seasons, autumn (May-June, 2001), winter (July-August, 2001) and summer (January–February, 2002). Autumn and summer records presented a high diversity of species (80.5 and 78 %, respectively), while only 46% of the species were present in winter (Figure 1).

## Taxonomic ranks

**Kingdom**: Animalia

**Phylum**: Chordata

**Class**: Chondrichthyes

**Order**: Chimaeriformes, Carcharhiniformes, Squaliformes, Torpediniformes, Rajiformes.

**Family**: Callorhinchidae, Scyliorhinidae, Squalidae, Torpedinidae, Rajidae

**Genera**: Callorhinchus, Schroederichthys, Squalus, Discopyge, Bathyraja, Dipturus, Psammobatis.

**Species**: *Callorhinchus callorhynchus*, *Schroederichthys bivius*, *Squalus acanthias*, *Discopyge tschudii*, *Bathyraja brachyurops*, *Bathyraja albomaculata*, *Bathyraja scaphiops*, *Bathyraja macloviana*, *Dipturus chilensis*, *Psammobatis scobina*, *Psammobatis normani*.

**Class**: Actinopterygii

**Order**: Anguilliformes, Gadiformes, Ophidiiformes, Pleuronectiformes, Perciformes, Scorpaeniformes.

**Family**: Congridae, Macrouridae, Moridae, Merlucciidae, Gadidae, Ophidiidae, Paralychthydae, Achiropsettidae, Scombridae, Centrolophidae, Stromateidae, Pinguipedidae, Carangidae, Serranidae, Bramidae, Cheilodactylidae, Bovichtidae, Nototheniidae, Scorpaenidae, Congiopodidae, Triglidae, Zoarcidae, Psychrolutidae.

**Genera**: Bassanago, Coelorhynchus, Salilota, Austrophycis, Macruronus, Merluccius, Micromesistius, Genypterus, Paralichthys, Mancopsetta, Scomber, Seriolella, Stromateus, Pseudopercis, Parona, Acanthistius, Brama, Nemadactylus, Cottoperca, Dissostichus, Patagonotothen, Sebastes, Congiopodus, Prionotus, Iluocoetes, Cottunculus.

**Species**: *Bassanago albescens*, *Coelorhynchus fasciatus*, *Salilota australis*, *Austrophycis marginata*, *Macruronus magellanicus*, *Merluccius hubbsi*, *Merluccius asutralis*, *Micromesistius australis*, *Genypterus blacodes*, *Paralichthys patagonicus*, *Mancopsetta maculata*, *Scomber japonicus*, *Seriolella punctata*, *Stromateus brasiliensis*, *Pseudopercis semifasciata*, *Parona signata*, *Acanthistius patachonicus*, *Brama brama*, *Nemadactylus bergi*, *Cottoperca gobio*, *Dissostichus eleginoides*, *Patagonotothen ramsayi*, *Pseudopercis*, *Sebastes oculata*, *Congiopodus peruvianus*, *Prionotus nudigula*, *Iluocoetes fimbriatus*, *Cottunculus granulosus*.

**Phylum**: Mollusca

**Class**: Cephalopoda

**Order**: Oegopsida

**Family**: Ommastrephidae, Onychoteuthidae.

**Genera**: Illex, Moroteuthis.

**Species**: *Illex argentinus*, Moroteuthis ingens.

## Spatial coverage

**Figure 2.**
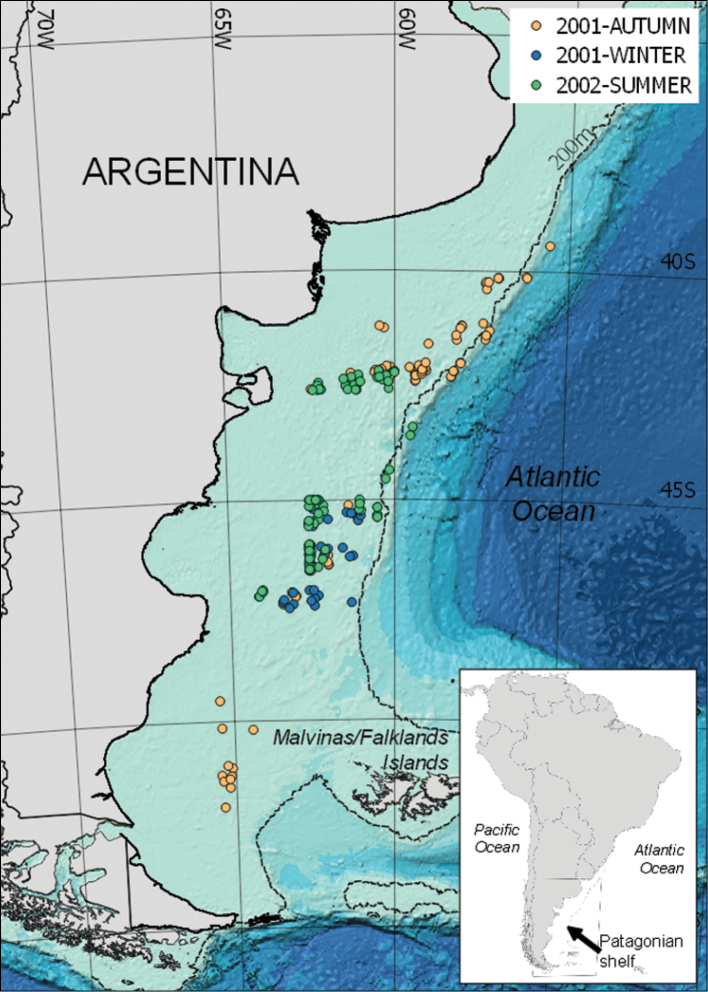
Spatial coverage (sampling locations).

**General spatial coverage**: The Argentine continental shelf, in the Atlantic margin of South America (Figure 2), is bounded by the line of the coast and the continental slope and is the most extensive submerged plain in the world, with an area of 930.000 km^2^ (Cousseau and Perrotta 2013). It is characterized by gentle slopes and low-relief. The shelf waters are of sub-Antarctic origin diluted by continental flows and modified by exchanges of mass and heat with the atmosphere. The main distribution of the species of this dataset is:

• The internal and external sectors of the shelf off the Province of Buenos Aires (35° S) to Patagonia (48° S).

• The southern part of the Patagonian-Fuegian shelf and Malvinas/Falkland Islands, along the outer shelf to the North, up to approximately 42° S.

• Deeper waters near the continental slope.

**Coordinates**: 52°S and 39°S Latitude; 65°W and 55°W Longitude.

**Temporal coverage**: May 11, 2001–June 26, 2001, July 21, 2001–August 31, 2001, January 11, 2002–February 27, 2002.

## Methods

### Study extent description

The sampling area was located on the Patagonian continental shelf (Figure 2), within 52 to 39°S and 65 to 55°W. This is the largest submerged plain in the Southern Hemisphere (930.000 km^2^), characterized by relatively shallow depth (mostly 150 m deep). Productive zones are associated with major water mass transitions, currents nutrient flow connected to upwelling and bathymetric features (Cosseau and Perrota 2013). The different water masses promote the recycling of nutrients accounting for its high productivity (Podesta et al. 1991, Carreto et al. 1995). The productivity of this area generates ‘hotspots’ of biodiversity and foraging of marine top predators is regularly observed (Croxall and Wood 2002, Campagna and Croxall 2003, Bastida and Rodríguez 2005). The specimens of this work were obtained through autumn (May-June, 2001), winter (July-August, 2001) and summer (January-February).

### Sampling description

Specimens of fish and squid (commercial target and by catch) were taken daily by commercial fishing vessels operating with bottom trawls during autumn (May-June, 2001, 51 catches), winter (July-August, 2001, 38 catches) and summer (January-February, 2002, 112 catches). The fishing company provided the associated data of each fishing haul: date, hour, location (decimal Latitude and Longitude of the position while pulling the net) and depth of the catch (the maximum depth reached by the net). Specimens were frozen on board, and identified at species level at the Ichthyology laboratory of Centro Nacional Patagónico, Puerto Madryn, Argentina. The taxonomical identification of species was made by the specialists Dr A. Gosztonyi and Dr M. Re and the scientific names and their current accurate spelling were also reviewed using suitable literature (Brunetti et al. 1998; Cousseau and Perrotta 2013) and the WoRMS web site (http://www.marinespecies.org/).

Sex (when possible) and morphometric measures were taken for each specimen: wet mass (g) and wet mass of viscera (g, empty stomach); total and standard length (cm) for fish; mantle, head and fin length and width (cm) for squid; left and right fin length (cm) and maximum fin width (cm) for skates.

### Method step description

Step1: Sampling locality and depth were recorded in each season.

Step2: Specimens were sent to the lab for species and sex identification and morphometric measurements.

### Datasets

The data is published on a Creative Commons Attribution Non Commercial (CC-BY-NC) 4.0 license.

### Dataset description

The star schema used to arrange the data has a DwC Event Core (seasons and catches) and two extensions: Occurrence (species) and ExtendedMeasurementOrFacts (individual morphometrics) (http://bdj.pensoft.net/articles.php?id=10989). The Darwin Core terms included in each file are:

Event core: type, eventID, parentEventID, samplingProtocol, eventDate, locationID, waterBody, locality, minimumDepthInMeters, decimalLatitude, decimalLongitude.

Occurrence (extension): modified, institutionCode, collectionCode, basisOfRecord, occurrenceID, catalogNumber, sex, occurrenceStatus, eventID, identifiedBy, scientificNameID, scientificName, kingdom, phylum, class, order, family, genus, specificEpithet, scientificNameAuthorship.

ExtendedMeasurementOrFacts (extension): occurrenceID, measurementType, measurementTypeID, measurementValue, measurementUnit, measurementUnitID, measurementDeterminedDate, measurementDeterminedBy.

**Object name**: Darwin Core Archive Demersal and pelagic species of fish and squid from the Patagonian shelf

**Character encoding**: UTF-8

**Format name**: Darwin Core Archive format

**Format version**: 1.0

**Distribution**: http://arobis.cenpat-conicet.gob.ar:8081/archive.do?r=argentina-fishes

**Publication date of data**: 2016-11-25

**Language**: English

**Licences of use**: This work is licensed under a Creative Commons Attribution Non Commercial (CC-BY-NC) 4.0 License (http://creativecommons.org/licenses/by-nc/4.0/legalcode).

**Metadata language**: English

**Date of metadata creation**: 2016-09-07

**Hierarchy level**: Dataset
